# Effects of endurance exercise under hypoxic conditions on the gastric emptying rate and intestinal cell damage

**DOI:** 10.1007/s00421-024-05523-1

**Published:** 2024-10-25

**Authors:** Sayaka Nomura, Daichi Sumi, Haruna Nagatsuka, Tomotaka Suzuki, Kazushige Goto

**Affiliations:** 1 Graduate School of Sport and Health Science, Ritsumeikan University, 1-1-1, Nojihigashi, Kusatsu, Shiga 525-8577 Japan; 2 Research Center for Urban Health and Sports, Osaka Metropolitan University, Osaka, Japan; 3 Research Fellow of Japan Society for the Promotion of Science, Tokyo, Japan

**Keywords:** Hypoxia, Post-exercise recovery, Gastrointestinal function

## Abstract

The present study examined the effects of gastric emptying rate and intestinal cell damage following a single session of endurance exercise under “hypoxic” or “normoxic” conditions at the same relative intensity. Eleven healthy males performed two trials on different days, consisting of a 60 min run on a treadmill at 70% maximal running velocity (vMax) while inspiring hypoxic (F_i_O_2_: 14.5%; HYP) or normoxic air (F_i_O_2_: 20.9%; NOR). The average running velocity was 11.4  ±  0.7 km/h in NOR and 10.8  ±  0.5 km/h in HYP, respectively. Venous blood samples were collected to evaluate plasma intestinal fatty acid binding protein (I-FABP) as an indicator of exercise-induced intestinal cell damage. The gastric emptying rate was determined by the ^13^C-sodium acetate breath test. Running velocities at 70% vMax and arterial oxygen saturation were significantly lower under HYP than NOR (*p* < 0.001). Peak heart rate and rating of perceived exertion during exercise did not differ significantly between the trials. Maximum ^13^C excretion time (an indication of the gastric emptying rate) was significantly delayed in the HYP (NOR: 38.5  ±  5.0 min, HYP: 45.5  ±  9.6 min; *p* = 0.010). Furthermore, the score of nausea increased slightly, but increased significantly after exercise only in the HYP (*p* = 0.04). However, exercise-induced changes in plasma I-FABP, adrenaline, and noradrenaline concentrations did not differ significantly between the two trials. These results suggest that endurance exercise under hypoxic conditions impairs digestive function in the stomach compared to exercise under normoxic conditions performed at the same relative intensity.

## Introduction

The gastrointestinal (GI) tract plays an important role in digestion and absorption for delivering nutrients to organs and skeletal muscles. GI function is evaluated by several variables, such as the gastric emptying (GE) rate, which affects nutrient digestion in the stomach, and the plasma intestinal fatty acid-binding protein (I-FABP) concentration, which is an indirect marker of damage to small intestinal cells (Horner et al. 2015; Chantler et al. 2021). In previous studies, endurance exercise above 70% of maximal oxygen uptake ($\dot{V}$O_2max_) delayed the GE rate and increased plasma I-FABP concentrations (Horner et al. 2015; Leiper. 2015; Ribeiro et al. 2021; Jonvik et al. 2019). A delayed GE rate impairs appetite and total energy intake, GI symptoms, and delays the time of appearance of ingested nutrients in the blood (Nair et al. 2009; Steege and Kolkman 2012; Kashima et al. 2018). Furthermore, van Wijck et al. (2013) suggested that the exercise-induced increase in plasma I-FABP is associated with the delayed appearance of ingested amino acids in the blood. Therefore, impaired GI function (i.e., delayed GE rate and increased plasma I-FABP concentration) may have an inhibitory effect on post-exercise recovery.

Although several factors are involved in the exacerbation of exercise-induced GI damage, the reduction in GI blood flow is thought to be a key factor (van Wijck et al. 2011, 2012; Kashima et al. 2017). As blood flow is predominantly distributed to the working muscles and skin during exercise, blood flow to the GI tract is reduced during exercise (Qamar and Read. 1987; van Wijck et al. 2012), which leads to hypoperfusion and ischemia of the intestinal cells and increased cell damage (Karhu et al. 2017; March et al. 2017). Van Wijck et al. (2011) reported that the increase in plasma I-FABP concentration following 60 min of endurance exercise at 70% of maximum workload (*W*_max_) is correlated with reduced GI blood flow. Additionally, 30 min of intermittent supra-maximal cycling exercise reduces GI blood flow and delays the GE rate (Kashima et al. 2017). Aside from the reduced blood flow in the GI tract, augmented sympathetic nervous system activity during exercise delays the GE rate. Endurance exercise reduces GI motility and delays the GE rate by activating sympathetic nervous system activity (Costa et al. 2017; Costa et al. 2019; Horner et al. 2015).

Exercise intensity and duration directly affect sympathetic nervous system activity and GI blood flow (Edwards et al. 2021; Mattin et al. 2020). In addition, environmental factors during exercise, including hypoxia, may be involved in exercise-induced GI dysfunction. Endurance exercise in a hypoxic environment has traditionally been used to improve endurance capacity (Czuba et al. 2011; Dufour et al. 2006; Ponsot et al. 2006; Zoll et al. 2006). Moreover, exercise under hypoxic conditions increases nitric oxide production compared to the same exercise under normoxic conditions (Casey and Joyner 2012), leading to augmented vasodilation with increased blood flow in working muscles (Casey and Joyner 2012; Joyner and Casey 2014). However, endurance exercise at 50% $\dot{V}$O_2peak_–65% $\dot{V}$O_2max_ under hypoxic conditions (inspired oxygen function (F_i_O_2_): 13.5–14.0%) significantly increases the plasma I-FABP concentration compared to the same exercise under normoxic conditions (F_i_O_2_: 20.9%) (Hill et al. 2020; Lee and Thake 2017). Katayama et al. (2010) revealed that endurance exercise-induced sympathetic nervous system activation is more profound under hypoxic than under normoxic conditions at relatively matched exercise intensity (50% of $\dot{V}$O_2peak_) (Katayama et al. 2010). Considering these previous studies, it is likely that exercise-induced GI dysfunction is facilitated in a hypoxic environment. However, the comparison of GE rate and intestinal cell damage following endurance exercise at “the same relative intensity” between normoxic and hypoxic conditions is still underexplored.

The purpose of the present study was to compare the GE rate and intestinal cell damage following endurance exercise between hypoxic and normoxic conditions under the same relative intensity.

## Methods

### Participants

Eleven healthy males participated in the present study (mean  ±  standard deviation (SD) age, 23.2  ±  1.1 years; height, 173.6  ±  3.4 cm; body mass, 66.3  ±  5.5 kg). At the onset of the present study, participants used to join regularly resistance training or endurance training 1 to 2 days per week. None of the participants were smokers, and none were injured or took medications or dietary supplements. Also, they did not have abdominal complaints during daily activities, history of gastrointestinal diseases/disorders, or abdominal surgery. All participants were born and lived at sea level. On the first visit to the laboratory, the participants were informed of the experimental procedures and the potential risks involved in the study, and written informed consent was obtained. The present study was conducted after approval by the Ethical Committee of Human Experiments at Ritsumeikan University (BKC-LSMH-2021–031), following the Declaration of Helsinki.

### Experimental overview

The participants visited the laboratory four times throughout the experiment. On the first two visits, they completed two bouts of maximal velocity (vMax) testing on a treadmill under normoxic (inspired oxygen fraction (F_i_O_2_) = 20.9%) or normobaric hypoxic conditions (F_i_O_2_ = 14.5%, equivalent to a simulated altitude of 3,000 m). Each trial was separated by about 3 days. On the third and fourth visits, the participants performed two different trials, consisting of a 60-min run on a treadmill under normoxia (NOR, F_i_O_2_ = 20.9%) or hypoxia (HYP, F_i_O_2_ = 14.5%). In each trial, running velocity was equivalent to 70% of normoxic vMax in the NOR and 70% of hypoxic vMax in the HYP to match the relative intensity between trials. Each trial was separated by at least a week, and the order of the trials was randomized.

After completing the 60-min exercise in each trial, participants rested for 90 min under normoxic conditions to investigate the GE rate and plasma I-FABP level during the post-exercise period. The normoxic or hypoxic air was provided during the trial via a specially designed mask with a tube connected to a hypoxic chamber system (Fuji Medical Science Co., Ltd. Chiba, Japan). The F_i_O_2_ used was blinded to the participants.

### Preliminary trials

Height was measured using a height analyzer (ST-2 M; Tanita Co., Tokyo, Japan). Body mass, body fat mass, and skeletal muscle mass were measured in 0.1 kg increments using a multi-frequency impedance analyzer (Inbody770; InBody Japan Inc. Tokyo, Japan). vMax was measured on a treadmill (Valiant; Lode, Groningen, the Netherlands) equipped with a fall prevention harness and an emergency stop device. After a warm-up exercise, the participants started running at 8 km/h, and the velocity was increased progressively by 2 km/h per min to 14 km/h. Then, the velocity was further increased by 0.6 km/h per min until exhaustion. The final speed (km/h) was defined as vMax.

### Main trials

During the 24 h before each trial, participants were asked to refrain from exercise, alcohol, and caffeine use. Participants were asked to consume a similar meal the day before the main trial and were allowed to drink 500 ml of water before arriving at the laboratory after waking up. The trial measurements were performed at the same time in the morning (8:00–12:00 am) following an overnight fast (water was consumed ad libitum) because nutrient intake immediately before the exercise has been shown to change GI barrier permeability, which may affect the experimental results (Sanford and Smyth 1974). After arriving at the laboratory with subsequent rest, the participants inhaled normoxic or hypoxic air for 5 min. Then, they started 60 min of running on a treadmill (Elevation series E95Ta; Life Fitness Corp.) under each condition. Running speeds were calculated as 70% vMax from the results of preliminary trials under the respective conditions. After completing 60 min of running, they rested for 90 min in a spinal position.

### Gastric emptying rate

The ^13^C-sodium acetate breath test was used to assess the GE rate (Ghoos et al. 1993; Braden et al. 1995). Before starting the exercise, baseline expired air was collected using a 1.3 L breath collection bag (Otsuka Pharmaceutical Co., Ltd., Tokyo, Japan). Five minutes after the end of the exercise, 100 mg of ^13^C-labelled sodium acetate (99 atomic%; Cambridge Isotope Laboratories Inc., Andover, MA, USA) was ingested with 100 mL of water. Seventeen breath samples were collected every 5 min during the 90-min post-exercise period. The ^13^CO₂/^12^CO₂ ratio in exhaled air was determined using an infrared spectrometer (POCone; Otsuka Pharmaceutical Co., Tokyo, Japan). All measurements were made in the supine position with the backrest at 45°. The production of CO_2_ per unit of body surface area was assumed to be 300 mmol/m^2^/h. Body surface area was calculated using the formula of Du Bois and Du Bois (Du Bois and Du Bois 1917). The times at which ^13^C-excretion/h indicated a maximum (i.e., *T*_max_ values) are closely correlated with the GE rates evaluated by scintigraphy (Ghoos et al. 1993; Braden et al. 1995).

### Blood variables

The participants arrived at the laboratory following an overnight fast (at least 10 h), and rested at least 15 min before the first blood was collected. Blood samples were taken immediately after exercise, 30 min after exercise, and 60 min after exercise. The blood samples were used to measure blood glucose, lactate, plasma adrenaline, noradrenaline, and I-FABP concentrations. Blood samples were taken from the antecubital vein and centrifuged at 4 °C and 3,000 rpm for 10 min to obtain the plasma. The plasma samples were stored at − 80 °C until analysis. Blood glucose and lactate concentrations were measured immediately after blood sampling using an automatic blood glucose analyzer (Free Style; Nipro Corp., Osaka, Japan) and a lactate analyzer (Lactate Pro; Arkray Co.). Plasma adrenaline and noradrenaline concentrations were measured by a clinical laboratory (SRL Inc.). Plasma I-FABP concentrations were analyzed using an enzyme-linked immunosorbent assay kit (R & D Systems, Minneapolis, MN, USA). The limit of detection was 6.21 pg/mL, assay range was 15.6—1,000 pg/mL (the samples were diluted before the assay). Assay was performed twice at all time points and the average value was the representative value. The intra-assay coefficient of variation was 2.2%.

### SpO_2_ and HR

Arterial oxygen saturation (SpO_2_) during exercise was measured after 30 min had elapsed using a finger pulse oximeter (Smart Pulse; Fukuda Denshi, Tokyo, Japan) placed on the tip of the forefinger. Heart rate (HR) was recorded every 1 min during exercise and the first 90 min after exercise using a wireless HR monitor (RCX5; Polar Electro Oy, Kempele, Finland).

### Subjective variables

Participants indicated their rating of perceived exertion (RPE) at the end of the 60 min of exercise using a 1–10 scale (Wilson and Jones 1991). Before and immediately after exercise, feelings of hunger, fullness, bloating, abdominal pain, nausea, and upset stomach were evaluated using a 1–10 cm visual analog scale (VAS). Severity was rated as mild (1–3), severe (4–5), or very severe (7–10) as described by McKenna et al. (2022).

### Statistical analysis

The sample size was calculated using G*Power version 3.1.9.7., and the result indicated that the minimum sample size was 10 (effect size = 0.8, *α* = 0.05, power (1 − *β*) = 0.80). We recruited 11 participants considering dropout.

Data are expressed as mean  ±  SD. Statistical analysis was performed using SPSS version 28.0.1.1 software (SPSS Inc., Chicago, IL, USA). Two-way repeated measures analysis of variance (ANOVA) was used to analyze the interaction (time × trial), the main effect of time (pre and post), and the main effect of trial (NOR and HYP). When ANOVA revealed a significant interaction or main effect, the Bonferroni adjustment was applied for the *post hoc* comparison. Results of vMax, running velocity, SpO_2_, RPE, and peak HR during exercise in the main trials were compared between the two trials using a dependent *t*-test. A *p*-value < 0.05 was considered significant. Effect sizes were determined using Cohen’s d for *t*-test. Effect size of *d* = 0.2 was classified as “small”, *d* = 0.5 was as “medium”, *d* = 0.8 was “large”, respectively (Cohen 1988).

## Results

### Running velocity, physiological variables and RPE

Table 1 shows running velocity, physiological variables and RPE in each trial. The vMax was significantly lower under the HYP than the NOR. Consequently, running velocity (70% vMax) was significantly lower during the main trials under the HYP than under the NOR. The HYP condition presented significantly lower SpO_2_ than NOR. Moreover, average HR during exercise was significantly higher in the HYP than in the NOR. However, no significant difference was observed between HYP and NOR for peak HR, peak RPE and average HR during post-exercise.

**Table 1:** Running velocity, physiological variables and RPE during the main trials

S	NOR	HYP	*p *value	Cohen’s *d* value
vMax (km/h)	16.3 ± 1.0	15.5 ± 0.7	< 0.001	0.933
Running velocity (km/h)	11.4 ± 0.7	10.8 ± 0.5	< 0.001	0.933
SpO_2_	96.0 ± 1.1	80.3 ± 3.2	0.001	6.465
Peak HR (bpm)	173.7 ± 12.9	177.1 ± 11.1	0.192	0.208
Average HR during (bpm)	166.1 ± 12.5	171.4 ± 10.2	0.033	0.471
Average HR after exercise (bpm)	83.2 ± 9.0	83.9 ± 8.8	0.661	0.073
RPE	6.0 ± 2.1	6.9 ± 2.2	0.085	0.424

Values are means  ±  SD

*NOR* exercise trial under normoxia, *HYP* exercise trial under hypoxia, *vMax* maximal velocity, *SpO*_*2*_ arterial oxygen saturation, *HR* heart rate, *RPE* rating of perceived exertion

### Gastric emptying rate

**Fig. 1:**
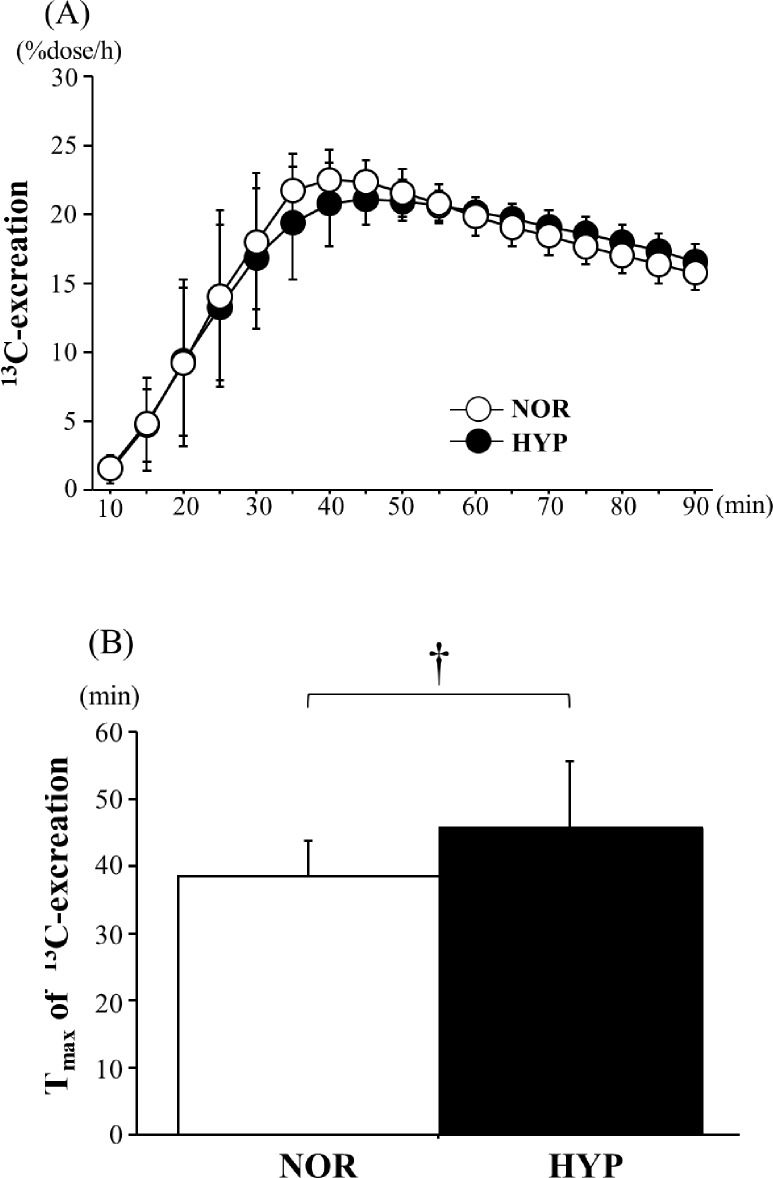
Changes in ^13^C-excretion (**A**) and T_max_ of ^13^C-excretion (**B**) after exercise. Values are means  ±  SD. *NOR* a trial with exercise under hypoxia, *HYP* a trial with exercise under hypoxia. ^†^Significant difference between trials

Figure 1 shows the changes in ^13^CO_2_ excretion during the post-exercise period. *T*_max_ was significantly higher in HYP than in NOR ((NOR: 38.5  ±  5.0 min, HYP: 45.5  ±  9.6 min; *p* = 0.010; *d* = 0.91), suggesting that the GE rate was significantly delayed under the HYP.

### Blood variables

Table 2 indicates the changes in blood glucose and lactate concentrations. The blood glucose concentration increased significantly after exercise in both trials, with no significant difference between trials. Blood lactate concentration increased significantly after exercise only in the HYP. Moreover, post-exercise blood lactate concentration was significantly higher under the HYP than under the NOR.

**Table 2:** Blood glucose and lactate concentrations before and after exercise

	NOR		HYP		*p *value		
	Pre	P0	Pre	P0	Interaction	Time	Trial
Glucose (mg/dL)	84 ± 4	97 ± 10^a^	81 ± 6	105 ± 18^a^	*p *= 0.035	*p *= 0.001	*p *= 0.334
Lactate (mmol/L)	1.7 ± 0.4	2.9 ± 1.5	1.9 ± 0.7	5.2 ± 2.3^ab^	*p *= 0.003	*p *= 0.003	*p *= 0.001

Values are mean  ±  SD

*NOR* a trial with exercise under hypoxia, *HYP* a trial with exercise under hypoxia, *Pre* pre-exercise, *P0* immediately after exercise

^a^*p* < 0.05 vs. Pre

^b^*p* < 0.05 between trials at the identical time point

**Fig. 2:**
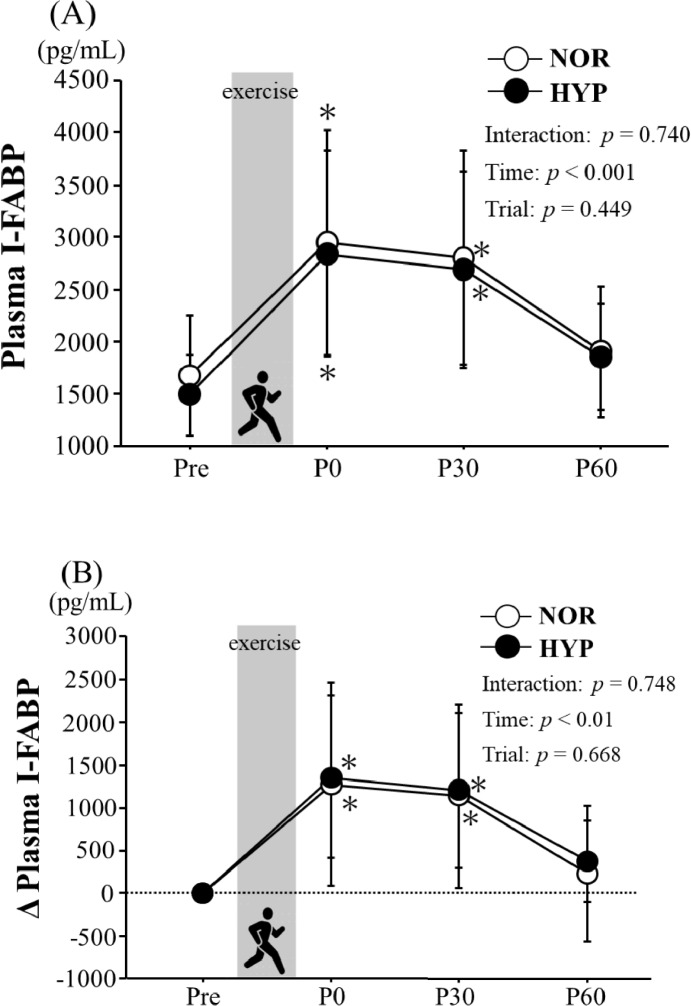
Changes in plasma I-FABP (**A**) and empty triangle plasma I-FABP concentrations (**B**). Values are means  ±  SD. *NOR* a trial with exercise under hypoxia, *HYP* a trial with exercise under hypoxia, *Pre* pre-exercise, *P0* immediately after exercise. *p < 0.05 vs. Pre

Figure 2 presents the changes in the plasma I-FABP concentration (A) and absolute change (Δ) in the plasma I-FABP concentration following exercise. Plasma I-FABP concentration increased significantly immediately after exercise in both trials. However, no significant difference was observed among the trials. Moreover, the Δ plasma I-FABP concentration did not differ significantly between the trials at any time point.

**Fig. 3:**
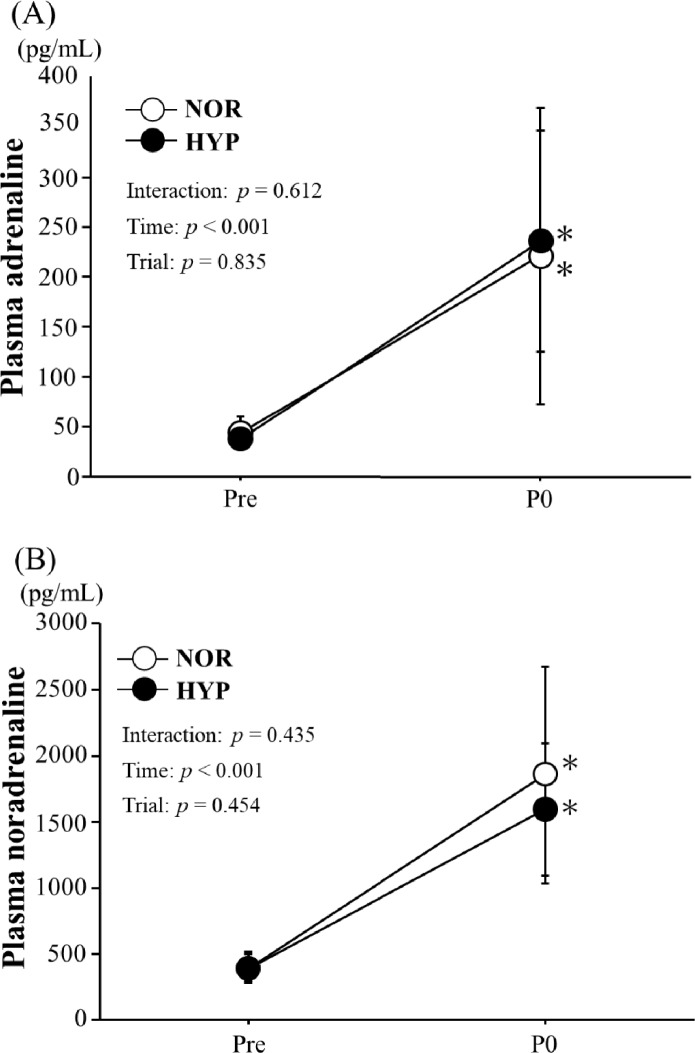
Changes in plasma adrenaline (**A**) and noradrenaline concentrations (**B**). Values are means  ±  SD. *NOR* a trial with exercise under hypoxia, *HYP* a trial with exercise under hypoxia, *Pre* pre-exercise, *P0* immediately after exercise. *p < 0.05 vs. Pre

Figure 3 shows the changes in plasma adrenaline and noradrenaline concentrations. Plasma adrenaline and noradrenaline concentrations increased significantly after exercise in both trials. However, these responses did not differ significantly between the trials.

### Scores of subjective variables

The scores of the subjective variables are shown in Table 3. The nausea score increased slightly and increased significantly after exercise only in the HYP. However, the appetite and GI symptom scores did not differ significantly between trials.

**Table 3:** Scores of subjective variables before and after exercise

	Pre	P0	*p *value		
			Interaction	Time	Trial
Hunger (cm)					
NOR	5.1 ± 3.1	3.2 ± 2.5	*p* = 0.349	*p* = 0.051	*p* = 0.926
HYP	5.5 ± 3.0	2.8 ± 2.6			
Fullness (cm)					
NOR	1.2 ± 1.8	1.9 ± 1.8	*p* = 0.460	*p* = 0.377	*p* = 0.697
HYP	1.7 ± 1.9	1.9 ± 2.2			
Bloating (cm)					
NOR	1.3 ± 1.2	1.1 ± 1.2	*p* = 0.404	*p* = 0.897	*p* = 0.186
HYP	1.6 ± 1.3	1.7 ± 1.3			
Abdominal pain (cm)					
NOR	0.8 ± 1.7	0.7 ± 1.0	*p* = 0.476	*p* = 0.908	*p* = 0.535
HYP	0.4 ± 0.6	0.7 ± 1.1			
Nausea (cm)					
NOR	0.4 ± 0.7	0.8 ± 0.9	*p* = 0.080	*p* = 0.040	*p* = 0.134
HYP	0.5 ± 0.9	1.6 ± 2.1^a^			
Upset stomach (cm)					
NOR	0.3 ± 0.5	0.8 ± 1.1	*p* = 0.746	*p* = 0.119	*p* = 0.989
HYP	0.4 ± 0.5	0.7 ± 1.3			

Values are mean  ±  SD

*NOR* a trial with exercise under hypoxia, *HYP* a trial with exercise under hypoxia, *Pre* pre-exercise, *P0* immediately after exercise

^a^*p* < 0.05 vs. Pre

## Discussion

The present study compared the endurance exercise-induced GE rate and intestinal cell damage under hypoxic and normoxic conditions. Although a similar comparison was conducted previously using matched exercise intensity (Hill et al. 2020; Lee and Thake 2017), we used relatively matched exercise intensity (i.e., 70% vMax) between the HYP and NOR trials. The main findings of the present study were that (i) the GE rate was delayed after 60 min of running exercise under the HYP trial compared to the same exercise under the NOR trial, and (ii) the increase in exercise-induced plasma I-FABP did not differ between trials.

Exercise-induced activation of the sympathetic nervous system delays the GE rate (Brown et al. 1994). Thirty minutes of submaximal endurance exercise at the same relative intensity (50% $\dot{V}$O_2peak_) under hypoxia (F_i_O_2_: 16.5%) causes further increases in HR and plasma adrenaline concentrations compared to the same exercise under normoxia (Katayama et al. 2010). In contrast, in the present study, HR and the exercise-induced increase in plasma adrenaline and noradrenaline concentrations did not differ significantly between trials. Therefore, it appeared that the delayed GE rate seen under the HYP was not associated with increased sympathetic nervous system activity. Another potential factor may be a decrease in GI blood flow. An exercise-induced decrease in the GI blood flow delays the GE rate (Kashima et al. 2017). Damage to small intestinal cells is mainly caused by reduced blood flow to the GI tract, leading to a higher plasma I-FABP concentration after exercise (Jonvik et al. 2019; Karhu et al. 2017; van Wijck et al. 2012, 2011). However, in the present study, no significant difference in the exercise-induced increases in plasma I-FABP concentration was found between trials. This finding suggests that the delayed GE rate under the HYP was independent of differences in GI blood flow.

Besides augmented sympathetic nervous system activity and reduced GI blood flow, the effect of increases in appetite-regulating hormones and blood lactate needs to be the focus. Camilleri (2019) showed that increases in blood peptide YY (PYY) secreted from the small intestine delay the GE rate, while higher blood ghrelin concentrations are related to a facilitated GE rate. Endurance exercise increases the plasma PYY concentration with a concomitant decrease in the plasma ghrelin concentration (Schubert et al. 2014). Moreover, exercise-induced changes in these hormones are augmented when exercising under hypoxic conditions (F_i_O_2_: 11.7–15.8%) (Bailey et al. 2015; Matu et al. 2017). While we were unable to evaluate plasma PYY or ghrelin concentrations following exercise, exercise-induced changes in these hormones may have been further facilitated under the HYP condition, which may have caused the delayed GE rate. Moreover, Silva et al. (2014) investigated the relationship between blood lactate concentration and the GE rate after 15 min of swimming exercise (using a loaded equivalent to 5% of their body weight) in rats. Consequently, the increase in blood lactate concentration immediately after exercise was related to the amount of residual gastric contents. Notably, administering sodium bicarbonate 40 min before the swimming exercise abolished the impaired effect of the stomach residue following exercise compared to the control condition without exercise, which was probably mediated by facilitating the buffering action of lactic acid. In human studies, high blood lactate concentrations following 30 min of resistance exercise are associated with a delayed GE rate (Kashima et al. 2018). In the present study, post-exercise blood lactate concentration was significantly higher in the HYP. Although the detailed mechanism remains unclear, it is plausible that blood lactate concentrations immediately after exercise under the HYP partially affected the delayed GE rate in the present study.

Two previous studies (Hill et al. 2020; Lee and Thake 2017) reported that increases in plasma I-FABP concentrations after endurance exercise are further augmented under hypoxia (F_i_O_2_: 13.5–14.0%) than under normoxia. Inconsistent results between these two studies and the present study (i.e., no difference in the increase in plasma I-FABP) may have occurred due to different exercise intensities. In the previous studies (Hill et al. 2020; Lee and Thake 2017), running speed was matched between the hypoxic and normoxic conditions. However, as VO_2max_ is lower under hypoxic than under normoxic conditions (Ofner et al. 2014; Wadley et al. 2006), relative exercise intensity would be higher during running at an identical velocity under hypoxia. In addition, blood flow in working muscle increases with exercise intensity (Joyner and Casey 2015). Therefore, exercise intensity affects the increase in plasma I-FABP (Edwars et al. 2021; Chantler et al. 2021), and the higher relative exercise intensity in the hypoxic environment may explain the augmented increase in plasma I-FABP in previous studies. In contrast, we used relatively matched exercise intensity (i.e., 70% vMax) between the NOR and HYP trials (NOR: 11.4  ±  0.7 km/h, HYP: 10.8  ±  0.5 km/h). Therefore, different methodologies may have affected the results. Moreover, the increase in core temperature during endurance exercise also affects the increase in plasma I-FABP concentration. This is due to an increase in skin blood flow to dissipate heat, with a concomitant increase in core temperature and a reduction in GI blood flow (Neufer et al. 1989). However, no difference in core temperature has been reported during endurance exercise between hypoxic and normoxic conditions (Lee and Thake 2017). In support of this idea, the sweating rate did not differ significantly between trials in the present study.

The GE rate and plasma I-FABP concentration results were inconsistent. A similar trend was reported by a previous study (Sumi et al. 2023). Moreover, inconsistent results are understandable because the GE rate and the increase in plasma I-FABP concentration reflect physiological changes in different areas. A delayed GE rate delays the appearance of blood glucose and amino acids in the blood following the post-exercise meal and attenuates appetite and food intake (Nair et al. 2009; Kashima et al. 2018). Thus, further research is needed to determine whether the delayed GE rate under the hypoxic condition affects the rate of appearance of glucose and amino acids in the blood and whether it affects subsequent exercise performance.

The present study has several limitations. We evaluated indirectly GI blood flow using plasma I-FABP concentration. In fact, exercise-induced plasma I-FABP elevations have been reported to reflect reduced GI blood flow (Karhu et al. 2017; van Wijck et al. 2011). However, since elevated plasma I-FABP concentration is an indirect marker, direct determination of blood flow in the GI tract using ultrasound equipment will be required in future. Moreover, the present study showed that endurance exercise in hypoxic environment slowed GE rate compared to normoxic environment. However, we were not able to assess the required time of appearance of ingested nutrients in the blood. To clarify the influence of reduced GI function on subsequent recovery, it would be effective to assess the appearance time of glucose and/or amino acid in the blood after meal ingestion. Also, carbohydrate intake during endurance exercise alters GE rate (Horner et al. 2015). Thus, future research may need to focus on the impact of different nutritional conditions on the post-exercise GE rate. Moreover, the data collection from athletes would be valuable since participants in the present study were physically active males. Although there were several limitations, it was notable to reveal that exercise-induced digestive dysfunction was facilitated in the hypoxic environment, even at the same relative intensity between two trials. This result would provide valuable information for designing nutritional strategies following endurance exercise under hypoxic environment.

In conclusion, the GE rate was significantly delayed under the hypoxic compared with the normoxic condition following endurance exercise at the same relative intensity. However, the exercise-induced change in plasma I-FABP concentration did not differ significantly between trials. These results suggest that endurance exercise in a hypoxic environment impairs digestive function in the stomach compared to exercise in a normoxic environment at the same relative intensity. As a practical application, it may be recommended to select a diet that is less stressful for digestion.

## Data Availability

All data are included in the manuscript, with no supplemental data.
